# Myelination Borrows a Trick from Phage

**DOI:** 10.1371/journal.pbio.1001626

**Published:** 2013-08-13

**Authors:** Roland G. Roberts

**Affiliations:** Public Library of Science, Cambridge, United Kingdom

[Fig pbio-1001626-g001]The 20,000–30,000 proteins encoded by our genomes all had to come from somewhere originally, and Nature is a great recycler. Although some sections of proteins have arisen recently by co-option of previously noncoding sequence, looking at most protein structures gives you a distinct feeling of déjà vu. Time and again handy functional modules appear in novel guises, doing sort of the same job, but in a different context. Two papers just published in *PLOS Biology* showcase a particularly spectacular example of such repurposing, where part of a protein from the tail of a bacteriophage (a bug's bug) reappears in a transcription factor that has a starring role in the electrical insulation of our nervous system.

**Figure pbio-1001626-g001:**
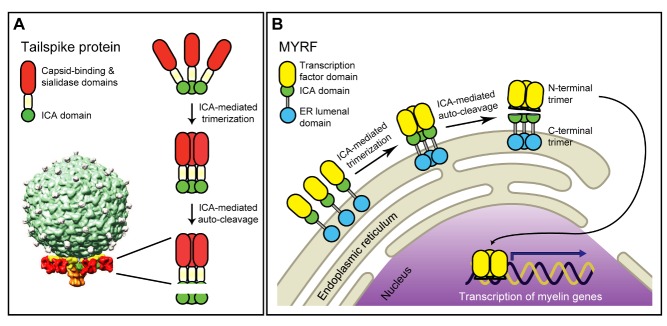
The same protein domain (ICA—green circle) mediates trimerization and self-cleavage in (A) the tail of a bacteriophage and (B) a transcription factor that drives insulation of nerves in the human brain. Image credit: Li, et al.

Transcription factors generally regulate genes by directly recognizing specific short sequence motifs in nearby regions of the genome. Because the genome is stored in the nucleus, the vast majority of transcription factors either reside in the nucleus, or commute in from the cytoplasm in response to a regulatory cue. However, there's an exclusive little coterie of transcription factors that start life partially embedded in a cellular membrane, and need to be liberated by proteolytic cleavage before they can set off to work in the nucleus. In most cases studied so far, this cleavage event is the lynchpin of the entire regulatory pathway—the SREBP proteins, for instance, are cleaved when cholesterol levels drop too low; ATF6 remains membrane-bound until misfolded proteins start to accumulate in the endoplasmic reticulum.

MYRF was identified in 2009 as a potential member of this select group, with what seemed to be the properties of both a transcription factor and a membrane protein—a DNA-binding domain reminiscent of yeast meiosis regulator Ndt80, plus a long run of oily amino acids characteristic of membrane-spanning anchors. MYRF is needed for the early steps of electrically insulating the axons of the neurons in our brain so that we can transmit nerve signals further, faster, and more efficiently. This process, known as myelination, is carried out by cells called oligodendrocytes that swaddle the delicate naked axons in fatty insulating sheaths. In the absence of MYRF, myelination stalls, with disastrous consequences for the axons.

Two papers now published in *PLOS Biology*—one from Helena Bujalka, Matthias Koenning, Ben Emery, and colleagues, and one from Zhihua Li, Yungki Park, and Edward Marcotte—reveal that MYRF is a self-cleaving, membrane-dwelling transcription factor with an intriguing pedigree. In each study, the crucial step was the recognition of a further patch of protein homology, just next to the yeast transcription factor look-alike. Amazingly, this region resembles the intramolecular chaperone autoprocessing (ICA) domain of bacteriophage tailspike proteins. Bacteriophages look rather like the Apollo lunar landing module, and the tailspikes are the bits that mediate touchdown on the bacterial surface. The ICA domain is known to help the tailspike protein bunch together in threes (to “trimerize”) and to self-cleave to form an active enzyme that snips away at the bacterium's cell wall.

Both groups then show conclusively that the ICA domain does exactly the same job in MYRF—it's essential for MYRF to trimerize and to cleave itself. The mechanism is presumably very similar, as mutating amino acids known to be important for phage protein self-cleavage or trimerization also affect the mammalian counterpart. The research groups then demonstrate that the cleavage is in turn needed to liberate the front half of the MYRF protein to make its way to the nucleus, leaving the back half behind at the endoplasmic reticulum.

Once in the nucleus, the business end of MYRF seeks out sites in the genome that contain a simple seven-base-pair DNA sequence motif. These MYRF-recognized sites are preferentially located near genes known to be important for oligodendrocytes, the cells that myelinate neurons in the brain. MYRF-bound regions containing these motifs function as powerful enhancers of transcription in an oligodendrocyte-like cell line and in primary oligodenrocytes, and mutation of either the DNA motif or the Ndt80-like region of MYRF wipes out this effect. Together, these two papers present compelling evidence that MYRF starts life in the endoplasmic reticulum, cleaves itself from a membrane-bound stalk, and shuttles to the nucleus, where it collaborates with other transcription factors such as Sox10 and Olig2 to initiate a program of oligodendrocyte development and nerve axon insulation.

Myelin is a vertebrate invention, though some other animals have come up with similar nervous system upgrades. But MYRF's intriguing story isn't limited to myelination—the eukaryotic tree of life is littered with MYRF relatives that share this crucial constellation of an Ndt80-like DNA-binding domain, a phage tailspike self-cleaving ICA domain, and a transmembrane tether. Sequence databases reveal that all vertebrates have two such proteins, and representatives are found in fruit flies, nematodes, sea anemones, and even the amoeba-like slime mold *Dictyostelium*. Some organisms with MYRF look-alikes lack nervous systems, let alone myelination, so these proteins can most likely be put to a range of uses.

An obvious puzzle is that MYRF seems to go to a great deal of trouble to undergo this liberation from the membrane, yet both research groups find that cleavage is constitutive (i.e., unconditional)—with other membrane-bound transcription factors the whole point of the cleavage is that it's exquisitely regulated, dependent on cellular conditions. Is this characteristic of MYRF merely because the conditions needed for cleavage are ever present in the experiments used by the authors? It's interesting that the nematode MYRF-related protein, pqn-47, seems to live in the endoplasmic reticulum—maybe its conditions for cleavage aren't yet met, and it's sitting there poised for action. And the slime mold version, MrfA, regulates an important developmental step in forming a stalk-like structure, presumably a time-critical process. Perhaps MYRF cleavage is conditional; we just don't know what that condition is yet.

The cute aspect of all of this, however, is MYRF's evolutionary journey, and the authors speculate as to whether this is a case of horizontal gene transfer. A parsimonious interpretation is that perhaps an ancient interaction between a phage particle and a single-celled eukaryote led to the insertion of part of a tailspike protein gene into a eukaryote transcription factor gene, and that the resulting (conditional?) self-cleavage was useful enough to be retained for the next billion years. Now that gene's descendants are telling slime molds when to form stalks and helping our brains to work fast enough to read papers about MYRF's role in myelination.

**Li Z, Park Y, Marcotte EM (2013) A Bacteriophage Tailspike Domain Promotes Self-Cleavage of a Human Membrane-Bound Transcription Factor, the Myelin Regulatory Factor MYRF. 10.1371/journal.pbio.1001624**

